# Does vitamin D supplementation improve ovarian reserve in women with diminished ovarian reserve and vitamin D deficiency: a before-and-after intervention study

**DOI:** 10.1186/s12902-021-00786-7

**Published:** 2021-06-21

**Authors:** Shahintaj Aramesh, Touran Alifarja, Ramin Jannesar, Parvin Ghaffari, Raziyeh Vanda, Fatemeh Bazarganipour

**Affiliations:** 1 Department of Gynecology and Obstetrics, Yasuj University of Medical Sciences, Yasuj, Iran; 2 Department of Pathology, Yasuj University of Medical Sciences, Yasuj, Iran; 3 Social Determinants of Health Research Center, Yasuj University of Medical Sciences, Yasuj, Iran

**Keywords:** Ovarian reserve, Vitamin D

## Abstract

**Objective:**

Evaluation of vitamin D supplementation on ovarian reserve in women with diminished ovarian reserve and vitamin D deficiency.

**Methods:**

The study is a before-and-after intervention study that was performed on women with diminished ovarian reserve referred to Shahid Mofteh Clinic in Yasuj, Iran. Eligible women were prescribed vitamin D tablets at a dose of 50,000 units weekly for up to 3 months. Serum levels of vitamin D and AMH were evaluated at the end of 3 months. Significance level was also considered *P* ≤ 0.05.

**Results:**

Our results have been showed there was a statistically significant difference in vitamin D levels of participants before [12.1(6.5)] and after [26(9.15)] the intervention (*P* < 0.001). Moreover, there was a statistically significant difference in serum AMH levels of participants before [0.50(0.44)] and after [0.79(0.15)] the intervention (*P=0.02* ).

**Conclusion:**

In conclusion, the results of the current study support a possible favorable effect of vitamin D on increase AMH expression by acting on the AMH gene promoter. Therefore, it is possible that vitamin D increases AMH levels without changing the antral follicle count/ovarian reserve.

## Introduction

Decreases in the quantity and quality of older eggs in women (typically in their mid to late 30s) are a natural physiological event called diminished ovarian reserve (DOR) [1]. Some women experience DOR much earlier and become infertile prematurely (pathological DOR). There is currently no “uniformly accepted definition of DOR” as stated by the American Reproductive Medicine Association (ASRM) Practice Committee in 2012 (p. 1412) [2]. However, in clinical practice, DOR has failed the ovarian abnormal storage test using various methods (such as low anti-Mullerian hormone [AMH], low antral follicle count [AFC], or, less frequently, a failed clomiphene citrate challenge test) among women who are still having regular periods [3, 4].

Antimullerian hormone (AMH) is a protein hormone and a known biomarker of ovarian reserve. AMH is principally expressed in growing granulosa cells of antral and pre-antral follicles [5]. AMH controls follicle growth by inhibiting their sensitivity to follicle-stimulating hormone (FSH) [6]. Therefore, decreased serum AMH levels in patients are an important indicator of ovarian reserve [5].

Vitamin D is a steroid hormone and has known effects on calcium and bone metabolism [7]. Previous studies have identified a functional element of vitamin D (VDRE) in the promoter region of the human AMH gene, indicating the potential direct effect of vitamin D on AMH expression [8]. Several in vitro and in vivo studies have studied the potential effects of vitamin D on ovarian function [9, 10]. Kinuta et al. [9] found that neutral Vitamin D receptor mice suffer from ovarian failure, which is characterized by impaired follicular growth. A recent meta-analysis evaluated the fertility outcomes of 2700 infertile women and found a significant relation of favorable outcomes with vitamin D-rich status [11]. It has been theorized that vitamin D affects ovarian follicles and may improve oocyte quality [10]. Based on the results of a recent systematic review and meta-analysis of vitamin D and AMH, more RCT are needed to explain the complex relationship between vitamin D and AMH [12].

To date, several observational and interventional studies have been performed to evaluate the relationship between serum levels of vitamin D and AMH, which presents very contradictory results. It should be noted that no intervention study was performed on the population with diminished ovarian reserve. Considering the above contradictory evidence and increasing interest in the role of vitamin D in female reproduction, our aim was to perform the first intervention studying the effect of vitamin D on ovarian reserve in an Iranian population.

## Methods

### Design and data collection

This is a before-and-after intervention study in 2018-2019 that performed on 51 women referring to referred to an infertility clinic, Yasuj, Iran. According to the inclusion criteria (vitamin D deficiency), we did not consider the control group for the present study and the design of this study was selected as a before- after interventional study.

Inclusion criteria were willing to contributing in study, patients’ age ranged from 18 to 40 years, being married, Iranian nationality, having no history of systematic diseases (diabetes, hypertension, known anemia, autoimmune or inflammatory disease and other diseases needing special diet), no smoking or drinking alcohol, no drugs intake, no specific gynecological diseases according to the approval of the gynecologist who performed the project (such as endometriosis, PCOS, ...), no history of surgery in the uterus and ovaries, no history of receiving chemotherapy or radiotherapy, AFC count lower than 10 in sonography at 3-10 days of menstrual cycle, AMH level lower than 1 ng/ml, serum Vit D level lower than 20 ng/ml. Exclusion criteria were unwilling to remain the study, hypercalcemia (defined as plasma calcium concentrations > 2.65 mmol/L), regular intake of vitamin D supplementation during 3 months prior to study inclusion.

According to the study of Dennis et al. (2017) [13] and α = 0.05, β =0.2, P1(increase of AMH in intervention group):0.87; p2(increase of AMH in control group): 0.45; the sample size were estimated at least 25 women for study.

### Measures

1. Demographic and reproductive information including age, occupation, education, smoking status, BMI and WHR were collected.
2. Serum 25-hydroxyvitamin D, Anti-Mullerian hormone and antral follicle counts evaluation:

The samples were centrifuged and stored at 20 °C. Vitamin D status was assessed by measuring serum levels of 25 OH-D in frozen samples by chemiluminescent immionoassay (CLIA) DiaSorin kit (DiaSorin Inc., Stillwater, MN, The LIAISON V R 25 OH Vitamin D TOTAL assay). The total imprecision coefficient of variance was 5.33% at a concentration level of 6.32 ng/mL and 4.96% at 37.71 ng/mL. The Endocrine Society has determined vitamin D insufficiency as serum levels between 20 to 30 ng/ml, and vitamin D deficiency as levels under 20 ng/ml. Moreover, regarding to the reference range of the kit, serum 25(OH)D levels < 20 ng/mL were considered deficient.

The samples were centrifuged and stored at -20 c. The assay of Antimullerianhormone was done by Ultra-Sensitive AMH/MIS ELISA kit of Ansh Labs company and use of automatic ELISAanalyser, elisys uno. The lowest amount of in a sample that can be detected with a 95% probability (n=24) is 0.023 ng/mL. The estimated minimum dose achieved at 20% total imprecision is 0.06 ng/mL. Reproducibility and total Coeficient variation of the AMH ELISA assay was 5.13, 6.03 and 4.46 percent at concentrations of 0.35, 0.72, 1.85 ng/ml respectively. The assay of vitamin D was done by 25 OH ELISA kit of Euroimmune company and use of automatic ELISA analyser, elisys uno. The inter assay precision was 7.8, 7 and 8.6 percent at concentrations of 16.6, 43.5 and 67.8 ng/ml respectively.

### Description of intervention

All patients received an oral dose of 50,000 IU vitamin D (Zahravi, Tabriz, Iran) per week for 3 months. After intervention, the AMH and vitamin D level were measured, again. In order to improve and confirm compliance, we asked participants to return full as well as empty medicine bottles at the end of the study.

### Statistical analysis

Data analysis were done using descriptive statistics (frequency, percent, median, IQR). The level of vitamin D and AMH was compared within groups using the signed-rank test. Mann-Whitney test was used for AMH level comparison between age categories (< 35 vs. >35y). We used descriptive statistics as well as the Kolmogorov–Smirnov test to analyze the distribution of data. We used non parametric statistical test due to non-normal distribution of data. The statistical program for Social Sciences (SPSS, version 21; SPSS, Chicago, IL). *P* values were set as 0.05 for all analyses. There were no missing values. Therefore, no missing imputation technique was used. Preparation of this manuscript was in accord with the STROBE guidelines for observational studies.

## Results

### Sample characteristics

Results Table 1 shows the characteristic of participants. As the findings in Table 1 show, the mean (IQR) age in the participants was 34(5) years. The main participants were in terms of education were in university and higher level (56.9%). Mean (IQR) BMI and WHR in participants were 27.55(5.93) and 0.85(0.11), respectively. Smoking was reported by 2% by the patient and 9.8% by the spouse. Mean (IQR) serum levels of AMH and AFC in participants before the intervention were 0.50(0.44) and 7(5), respectively.

**Table 1:** Characteristics of participants

**Variables**	
**Education**^a^	
Guidance school or less	3 (5.9)
Completed high school	19 (37.3)
College	29 (56.9)
**Age (years)**^b^	34 (5)
**Job**^a^	
Housewife	18 (35.8)
Employment	33 (64.7)
**BMI (kg/m2)**^b^	27.55 (5.93)
**WHR**^b^	0.85 (0.11)
**Using cigarette by patient**^a^	1 (2)
**Passive smoking**^a^	5 (9.8)
**AFC**^b^	7 (5)

^a^N(%); ^b^Median (IQR)

### Vitamin D and AMH level after intervention

Based on the results of sign test, there was a statistically significant difference in vitamin D levels of participants before [12.1(6.5)] and after [26(9.15)] the intervention (*P* < 0.001).

Based on the results of sign test, there was a statistically significant difference in serum AMH levels of participants before [0.50(0.44)] and after [0.79(0.15)] the intervention (*P* =0.02) (Table 2).

**Table 2:** Comparison AMH and vitamin D level before and after intervention

Variable	Before	After	*P* value*
AMH (ng/ml)^a^	0.50 (0.44)	0.79 (0.15)	0.02
Vitamin D (ng/ml)^a^	12.1 (6.5)	26 (9.15)	*P* < 0.001

*Z test; ^a^Median (IQR)

According to the results of Mann-Whitney test, there was no statistically significant difference in serum AMH levels of participants after the intervention based on age (*P* = 0.13).

## Discussion

The results of our single arm uncontrolled study showed that the serum level of vitamin D in patients with vitamin D deficiency increased significantly after the intervention and consequently their serum AMH increased significantly after the intervention. The available evidence regarding vitamin D and AMH levels is contradictory [12]. Vitamin D has been shown to regulate AMH levels in vitro directly via the AMH promoter and indirectly by regulating granulosa cell count and AMH expression in ovarian follicle culture [14, 15]. Contrary to the consistency of in vitro studies, the evidence for an association between vitamin D and AMH in women is controversial. Based on latest systematic review, the most cross-sectional studies failed to find a significant relationship between vitamin D level and AMH [12]. In contrast, a prospective study involving PCOS women found an association between vitamin D deficiency and decreased serum AMH levels [16]. In addition, the positive effects of vitamin D on AMH levels decreased in a prospective study including infertile women with diminished ovarian reserve [17]. Various reasons can underlie inconsistent findings in cross-sectional studies. Heterogeneity in the studied populations may indicate some inconsistent data reported, as some studies examined women with normal infertile ovulation, while others found women with PCOS or reduced ovarian reserve and others observed especially in infertile women. In addition, individual levels of vitamin D are affected by race / ethnicity, geographic area, and season (sun exposure) and have been suggested to play a role in differences in ovarian reserve [18]. In addition, differences in population size may be negative findings because some studies had a small number of patients [19–25] and did not have sufficient power to detect small but significant associations between vitamin D and AMH.

To best on our knowledge, there are only two small RCTs that examine the effects of vitamin D on AMH levels in women. In a study of infertile PCOS women with vitamin D deficiency, participants received 50,000 IU vitamin D / week (*n* = 17) or placebo (*n* = 17) for 8 weeks. The a significant decrease in AMH levels in the vitamin D group compared to placebo was found [26]. Dennis et al. performed RCTs in 49 young women with regular menstruation to evaluate the effects of vitamin D (50,000 IU, taken on the first day of the menstrual cycle) versus placebo on AMH levels over the next week. They found a significant increase in AMH levels in the week following vitamin D supplementation [27]. Although these results may seem contradictory at first, this contradiction can be resolved by considering the different follicular contexts and AMH status of women with PCOS. Women with PCOS typically have abnormal serum AMH levels, which reflect the large number of small antral ovarian follicles captured [28]. Elevated AMH levels in their cases are associated with severe PCOS manifestations [29]. In various studies, vitamin D supplements have also been shown lead to an improvement in the clinical manifestations of PCOS [30, 31]. Therefore, the reduction associated with AMH is not surprising but, most likely, reflects the improvement in folliculogenesis and ovulation status in these population.

Our study has different strengths compared to previous studies. First of all, In addition, AMH and vitamin D were measured in 1 day, indicating the true status of both hormones, while in the vast majority of the above studies, stored blood samples were used [15, 27, 32].

But we must highlight some limitations, which must be taken into account when interpreting our results. First, the sample size of the participants was relatively small. Second, given that the vast majority of patients in our study included Iranians, ethnicity was not assessed for vitamin D status. Third, we omitted the measurement of vitamin D binding protein, as none of the previous studies evaluated vitamin D binding protein, and vitamin D 25-OH is an accepted biomarker for vitamin D [33]. Morover we assess only did the serum level of AMH as an important marker in women with diminished ovarian reserve in relation to vitamin D 25-OH, like some previous studies. In addition, we did not evaluate the dietary patterns of the study participants. This limitation may affect our results, as certain diets, such as the Mediterranean diet, have been shown to be associated with circulating vitamin D concentrations [34]. Finally, according to the inclusion criteria (vitamin D deficiency), we did not consider the control group for the present study and the design of this study was selected as a before- after interventional study so it is not clear if the vitamin D intervention was responsible for the change.

In conclusion, allowing for the limitations mentioned above, the results of the current first interventional study in women with diminished ovarian reserve and vitamin D deficiency support a possible favorable effect of vitamin D on increase AMH expression by acting on the AMH gene promoter. Therefore, it is possible that vitamin D increases AMH levels without changing the antral follicle count/ovarian reserve. However, further adequately powered RCTs are of clinical importance to clarify the potential positive effects of vitamin D on ovarian reserve.

## Data Availability

The primary data for this study is available from the authors (SHA) on direct request.

## Abbreviations

AMH: Antimullerian hormone
CLIA: Chemiluminescent immionoassay
